# Physics of Majorana modes in interacting helical liquid

**DOI:** 10.1038/srep30569

**Published:** 2016-07-27

**Authors:** Sujit Sarkar

**Affiliations:** 1 Poornaprajna Institute of Scientific Research, 4 Sadashivanagar, Bangalore 5600 80, India

## Abstract

As an attempt to understand and search for the existence of Majorana zero mode, we study the topological quantum phase transition and also the nature of this transition in helical liquid system, which appears in different physical systems. We present Majorana-Ising transition along with the phase boundary in the presence of interaction. We show the appearance of Majorana mode under the renormalization of the parameters of the system and also the topological protection of it. We present the length scale dependent condition for the appearance of Majorana edge state and also the absence of edge state for a certain regime of parameter space.

In recent years, Majorana fermion physics and the topological state of matter have been the focus of intense research in condensed matter physics^1^,^2^,^3^,^4^. Experimental observation of Majorana zero mode is not unambiguous identification of Majorana mode^5^,^6^,^7^,^8^,^9^,^10^. Therefore, the subject of Majorana mode remains open.

This field of physics initiates several ideas and routes to understand and predict the existence of Majorana zero mode. Thus this field motivates us to search for and find the Majorana zero mode physics in interacting helical liquid, which appears in different quantum many body systems^11^,^12^,^13^,^14^,^15^,^16^,^17^,^18^,^19^,^20^,^21^,^22^,^23^,^24^,^25^,^26^,^27^,^28^,^29^,^30^,^31^,^32^. The physics of helical liquid is quite interesting^13^. It is generally originated from the quantum spin Hall effect in a system with or without Landau levels. In the quantum spin Hall effect, the left movers in the edge are connected with the down spin and the right movers with the up spin and the transport process is quantized. This physics is generally termed as a “helical liquid” which describes the connection between the spin and momentum. It does not break the time reversal invariance which occurs in chiral Luttinger liquid. One important consequence of the topological insulator/helical spin liquid system is the existence of edge state, which is the gapless excitation and remains the same in presence of interactions.

In this study, we focus on the Majorana-Ising transition and the existence of Majorana edge state under the renormalization of proximity induced superconductivity (Δ) and applied magnetic field (*B*) in helical liquid system. The other goal of this study is to find out the length scale dependent condition for the appearance of Majorana fermion mode physics in helical liquid systems especially the absence of zero energy Majorana fermion mode in the deep repulsive regime of interactions, which are entirely new studies and results in the literature for this system.

One can write the Hamiltonian for the low energy collective excitation in one dimensional system as


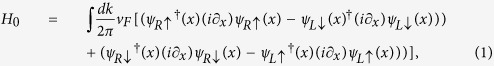


where *ψ*_*R*↑_(*x*) and *ψ*_*L*↓_(*x*) are the field operators for spin up right moving and spin down left moving electrons respectively. The term within the parenthesis are the respective Kramer’s pair. One of these Kramer’s pairs is in the upper edge and the other one in the lower edge of the system. The total fermionic field of this system is, 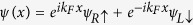. This is the most basic picture of a helical liquid, where the spin is determined by the direction of the particle.

The non-interacting part of the helical liquid for a single edge in terms of fermionic field is 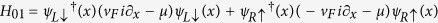. We use *H*_01_ during the derivation of Renormalization Group equations (please see the method section). The first two terms are from the *H*_01_. The relation between *H*_0_ and *H*_01_ is the following: *H*_0_ represent two Kramer’s pairs for the both upper and lower edge of the system, where *H*_01_ represent the single Kramer’s pair for a single edge of this two dimensional system. The physics is the same for the both edge of the topological insulator. Therefore, we consider a single edge Hamiltonian *H*_01_.

Here we consider a low-dimensional quantum many body system of topological insulator in the proximity of s-wave superconductor and an external magnetic field along the edge of this system. The additional part in the Hamiltonian is


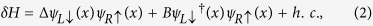


where Δ is the proximity induced superconducting gap and *B* is the applied magnetic field along the edge of the sample. It introduces the gap in the spectrum of the edge state. It causes a spin flip process which requires one to flip the momentum and hence to exchange a right movers with a left mover. The applied magnetic field align the physical spin in the direction parallel to the magnetic field^16^,^23^.

Now we consider the generic interaction, considering the two particles having forward and umklapp scattering as


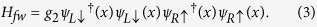


The analytical expression for umklapp in a conventional form can be expressed as^23^.


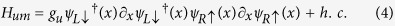


Therefore, the total Hamiltonian of the system is *H* = *H*_0_ + *H*_*fw*_ + *H*_*um*_ + *δH*. Now we can write the above Hamiltonian as *H*_*XYZ*_ = ∑_*i*_*H*_*i*_ (up to a constant)^16^, where


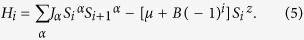


and *J*_*x*,*y*_ = *J* ± Δ > 0, *J* = *v*_*F*_ and *J*_*z*_ > 0.

The bosonized form of this model Hamiltonian is (for detail, please see method).


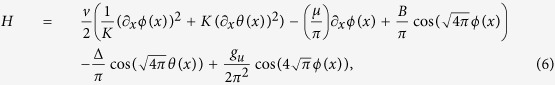


where *θ*(*x*) and *ϕ*(*x*) are the dual fields and *K* is the Luttinger liquid parameter of the system. The first and second term of the above equation are the bosonized version of Hamiltonian *H*_01_. The general interactions of this model Hamiltonian are the same as that given in ref. 16.

The Abelian bosonization study of this model Hamiltonian^16^ is not physically consistent for the following reason: It is very clear from the continuum field theoretical study that our model Hamiltonian contains two strongly relevant and mutually nonlocal perturbations over the Gaussian (critical) theory. In such a situation, the strong coupling fixed point is usually determined by the most relevant perturbation whose amplitude grows up according to its Gaussian scaling dimensions and it is not much affected by the less relevant coupling terms. However, this is not the general rule if the two operators exclude each other. In this case, the interplay between the two competing relevant operators (here Δ and *B* are the two competing relevant operators, which are related with dual fields *θ*(*x*) and *ϕ*(*x*)) can produce a novel quantum phase transition through a critical point or a critical line. Therefore, the present study based on RG equations will give us the appropriate results for the topological state of the system over the previous studies^16^,^28^.

## Results

### Majorana-Ising transition and nature of phase transition

Our starting Hamiltonian is *H*_2_ = *H*_0_ + *δH*. We recast the fermionic field in terms of the Majorana fields as, 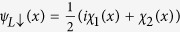 and 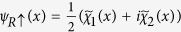. The total Hamiltonian, *H* = *H*_0_ + *δH*, becomes


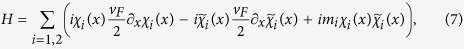


where 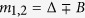 (here Δ > 0). At Δ = *B*, one of the two Majorana fermion modes becomes gapless which is the signature of bulk Majorana-Ising quantum phase transition.

It is well known that the critical theory is invariant under the rescaling. Then the singular part of the free energy density satisfies the following scaling relations.

**Figure 1:**
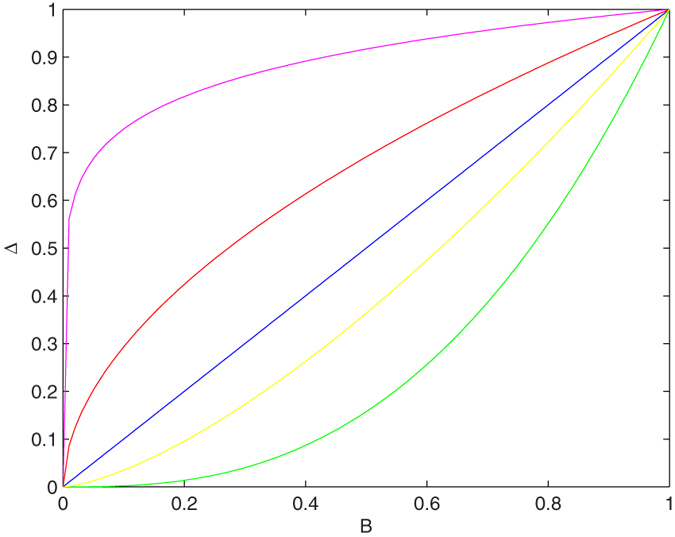
Phase boundary between the two different phases Δ and *B*. The color blue, magenta, red, green and yellow are for *K* = 1, 0.55, 0.75, 1.5, 1.2 respectively.

*f*_*s*_[Δ, *B*] = *e*^−2*l*^*f*_*s*_[*e*^(2−1/*K*)*l*^Δ, *e*^(2−*K*)*l*^*B*]. The scale *l* can be fixed from the following analytical relation, 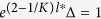. Finally, after few steps of calculation, we arrive at the following relation: *f*_*s*_[Δ, *B*] = Δ^2/(2−1/*K*)^*f*_*s*_[1, Δ^−(2−*K*)/(2−1/*K*)^*B*] and the equation for phase transition is 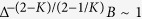. The phase boundary between these two quantum phases can be obtained by using the above relation. When *K* = 1(non-interacting case), the phase boundary relation based on the exact scaling relation is Δ = *B*. The authors of ref. 32 have done a very time relevant study for the prediction of phase boundaries for more general interactions in superconducting wire. They have found that the phase boundaries between the topological and non-topological phases, which are always straight lines. In the present study, based on the exact scaling relation, we observe that the separation between the topological and non-topological phase is straight line only for the non-interacting (*K* = 1) case otherwise the phase boundaries for repulsive and attractive interactions are curved as guided by the exact scaling relation. This exact scaling relation between the Δ, *B* and *K* is the non-linear one. Phase boundaries shift due to the presence of repulsive (*K* < 1) and attractive (*K* > 1) interactions on this phase diagram, which is presented in Fig. 1. It reveals from this study that the topological phase region suppress for the repulsive interactions and favors for the attractive interactions.

Now we explain physically about the origin of the shifting of the phase boundaries in presence of interactions from the non-interacting one. Generally, one can consider the interaction between the Majorana fermions as 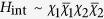. In the mean field level, one can consider the following approximation, 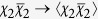. Thus it is clear from the mean-field analysis that one can absorb the effect of interaction as a redefined mass in the system (third term of equation 7) which shifts the phase boundary. Apart from this study on the phase boundaries, we also study the nature of the quantum phase transitions (please see below). This detail study based on exact scaling relation for the phase boundaries is absent in the literature for this model Hamiltonian System.

#### Nature of Quantum Phase Transition

To find the nature of quantum phases during the topological quantum phase transition, we do a toy model analysis. We consider the simple case when the umklapp term is absent at the point Δ = *J*. Then the complete Hamiltonian reduces to transverse Ising model as


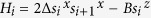
. When *B* < Δ, the discrete Ising symmetry is spontaneously broken which yields a doubly degenerate ordered phase. Then it induces the superconducting gap state which forms the Majorana fermion mode excitations at the edge of the system. For *B* > Δ, the magnetic field induces the ferromagnetic state along the direction of the magnetic field.

### Renormalization Group study to predict the Majorana mode

**Figure 2:**
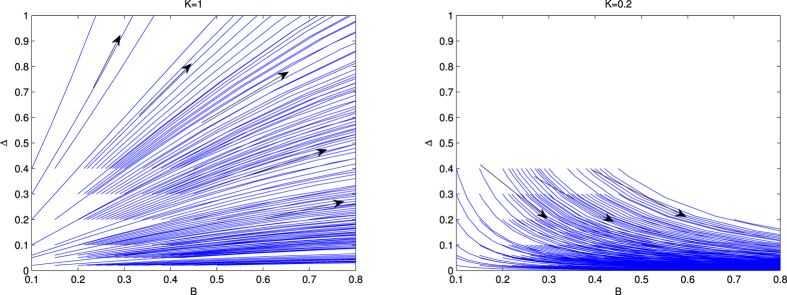
RG flow of Δ with B, in the absence of umklapp scattering. The left panel (Fig. 2A) is for *K* = 1 and the right panel (Fig. 2B) is for *K* = 0.2.

Figure 2, consists of two panels, the left one (Fig. 2A) is for *K* = 1, and the right one (Fig. 2B) is for *K* = 0.2. In each panel, we present the RG flow diagram for Δ with *B*. It reveals from our study of left panel (Fig. 2A, *K* = 1) that both the couplings (Δ and *B*) are flowing off to the strong coupling phase. Here both the coupling terms are relevant. In the right panel (Fig. 2B, *K* = 0.2), i.e, when the system is in the strongly repulsive regime, our study reveals that the coupling term, Δ is flowing off to zero. In this case there is no existence of Majorana fermion mode in the system. The magnetic field induced ferromagnetic phase dictated by the direction of magnetic field is the only phase that exists in the system^23^.

Therefore, it is clear from the study of Fig. 2, that the Majorana fermion mode disappears only in the presence of very strong repulsion. Otherwise, the Majorana fermion mode is robust, i.e., topologically protected from weak and intermediate values of strong repulsion.

**Figure 3:**
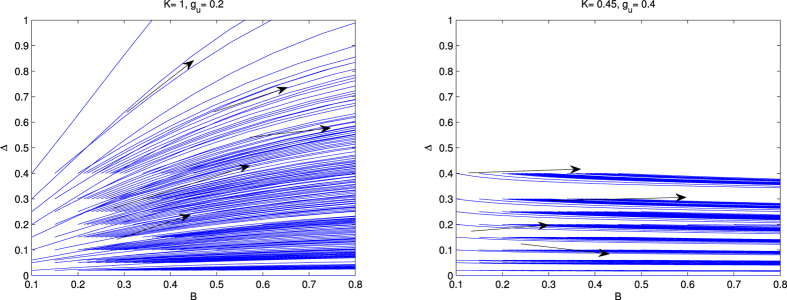
RG flow lines of Δ with *B*. The left panel (Fig. 3A) is for *K* = 1, *g*_*u*_ = 0.2 and the right (Fig. 3B) panel is for *K* = 0.45, *g*_*u*_ = 0.4.

In Fig. 3, we present the result of the variation of Δ with *B* in the presence of umklapp scattering. This figure consist of two panels. In the left panel (Fig. 3A), *K* = 1 and *g*_*u*_ = 0.2 and the right panel (Fig. 3B) *K* = 0.45 and *g*_*u*_ = 0.4. The behavior of the RG flow is the same for each panel as that of the absence of *g*_*u*_. Therefore, it is clear from this study that the Majorana zero mode is robust, i.e., topologically protected in the presence of umklapp scattering due to their topological protection. The authors of ref. 16, have studied the effect of umklapp scattering by using the Abelian bosonization method. But the present Hamiltonian consists of the three sine-Gordon coupling terms (last three terms of eq. 6) not the single one. At the same time they are the functions of *ϕ*(*x*) and *θ*(*x*). The fields *ϕ*(*x*) and *θ*(*x*) are the mutually exclusive, i.e., the minima of one is not the minima of other. As we have already discussed (after eq. 6) that for these case to get the correct physical pictures one has to be done RG study. Thus the results what we obtain based on RG analysis is more correct and physical.

### Length scale dependent Majorana edge state

The Majorana edge state survives at the edge of the system if it satisfies the condition for a system with length *L* as 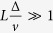, where *v* is the velocity of collective mode of system and Δ is superconducting gap. Otherwise, there is no Majorana edge state, i.e., the topological state of the system is absent^30^,^31^.

Now we discuss physical origin for the condition of appearance of Majorana fermion edge mode very briefly: In a nanowire or at the edge of topological insulator, where the helical spin liquid form in a edge, the Majorana edge state appears as the particle-hole bound state at the both end of the wire or edge with localization length 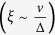. The overlap of the Majorana wave functions is proportional to *e*^−*L*/*ξ*^. The existence of the Majorana fermion zero mode requires that the wave function overlap should be vanishingly small, that finally implies the condition which we have mentioned above.

**Figure 4:**
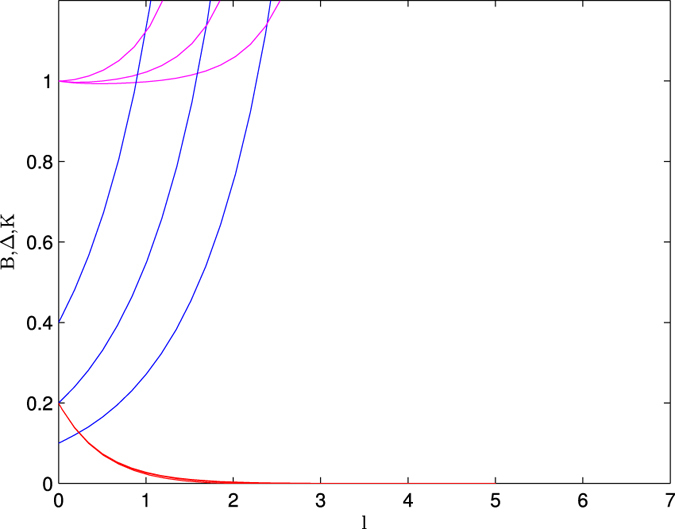
The variation of Δ, *B* and *K* with length scale (*l*). The curves with blue, pink and red lines are respectively for the Δ, *K* and *B*. Δ = 0.1, 0.2, 0.4; *B* = 0.2, *K* = 1.

In Fig. 4, we present the results of the study of Δ, *B* and *K* with the length scale (*l*) and three initials values of Δ = 0.1, 0.2, 0.4 with the initial value of *B* = 0.2. These values are same as the values of above three curves. The study of the left panel (*K* = 1) shows the existence of Majorana edge state in the system. For all values of Δ show that it increases very rapidly with the length scale which satisfy the condition for the existence of Majorana edge states. At the same time, we observe that *B* decreases rapidly, i.e., the zero mode Majorana edge state protected. Thus it is clear from this study that the system is in always topological state with zero mode Majorana edge state when the system is non-interacting.

**Figure 5:**
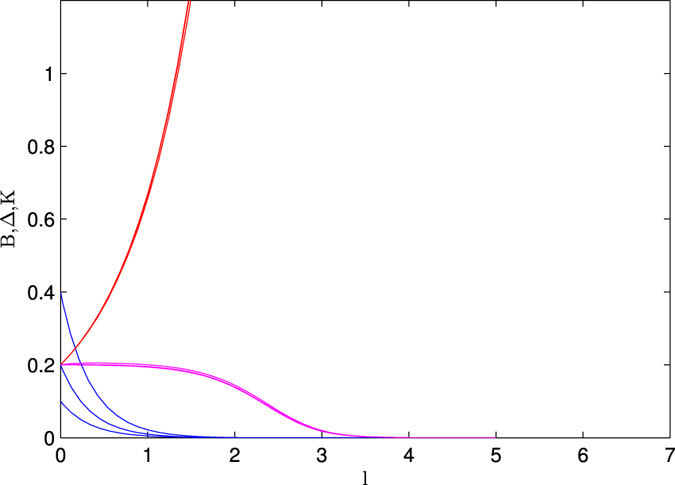
The variation of Δ, *B* and *K* with length scale (*l*). The curves with blue, pink and red lines are respectively for the Δ, *K* and *B*. Δ = 0.1, 0.2, 0.4; *B* = 0.2. Left panel (Fig. 5A) is for *K* = 0.45, the right panel (Fig. 5B) is for *K* = 0.2.

In Fig. 5, we present the results of the study of Δ, *B* and *K* with the length scale under RG process in presence of repulsive interactions (*K* = 0.2). It is clear from this study that for all values of Δs decrease very rapidly with length scale. And the condition for the existence of Majorana fermion mode is violating for this strong repulsive interaction. In presence of interaction, the condition for the absence of zero energy Majorana fermion mode become 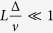, for this case Majorana modes hybridize strongly and shift from the zero energy mode. Therefore, it is very clear from our study that the strong repulsive interaction strongly suppress the possibilities of zero mode Majorana edge state. Note that our study is completely an independent study with new results for this model Hamiltonian to predict the length scale dependent topological state which is absent in refs 16 and 28.

### Effect of Chemical Potential

Here we study explicitly, how the presence of chemical potential affect the different quantum phases for the different regime of interactions of this system.

At first we consider only the presence of *B* (*g*_*u*_ = 0, Δ = 0). To study this effect, we consider the following transformation, 2*ϕ* → 2*ϕ* + *δ*_1_*x*, where 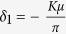. This transformation eliminates the term ∂_*x*_*ϕ*(*x*) from the bosonized Hamiltonian (equation (6)) but this transformation leads to a spatially oscillating term, i.e., 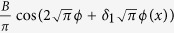. For this situation system shows the commensurate to incommensurate transition. This term is quickly oscillating and averages out to zero when *δ*_1_*a* ≫ 1, which reflects the competition between the *μ* and *B*. As a result of this competition, the RG flow in B has to be cut-off when 2*δ*_1_(*l*) ~ 1.

Now we consider the case when both *B* and Δ are non-zero. To study this effect explicitly, we use a RG equation of *δ*_1_. To the lowest order in *B*, Δ and *δ*_1_, the RG flows of *δ*_1_ is 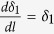
29. When all the perturbations are relevant, they flow to the strong coupling phase under RG transformation. If the coupling *B*(*l*) reaches to the strong coupling phase before *δ*_1_(*l*)*a* reaches to one, the phase of the system is ferromagnetic phase under the condition *δ*_1_(0)*a* ≪ *B*(0)^1/(2−*K*)^. When Δ(*l*) term reaches to the strong coupling phase, then the system shows the existence of Majorana edge state.

In the presence of umklapp term, the system posses an oscillatory term 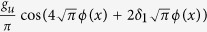. If the *g*_*u*_(*l*) term reaches to the strong coupling phase earlier than *B*(*l*), then the system is in the ferromagnetic phase.

Therefore, it is clear from the above study based on the RG equation of *δ*_1_ that the different quantum phases of this system dominates in presence of chemical potential in the different regime of the interaction space. This detail study of the effect of *μ* is absent in the previous studies, refs 16 and 28.

## Discussions

We have found Majorana-Ising transition for helical liquid based on the exact analytical expression for phase boundary and also explained the nature of this transition. We have carried out the renormalization group study of the interacting helical liquid in the presence of proximity induced superconductivity and applied magnetic field along the edge direction to predict the Majorana mode in the presence of interaction and umklapp scattering. We have also found the topological protection in the system. We have studied the length scale depended condition for the existence of Majorana edge state, which is entirely a new results in the literature for this system. We have also found a regime of parameter space for repulsive regime where there is no evidence of Majorana edge state. Our renormalization group study has yielded the correct analytical and physical explanation for the topological state of matter under the variation of interaction parameters of the system.

## Methods

### Bosonized Hamiltonian

In the bosonization process, one can express the fermionic field of one dimensional quantum many body system as 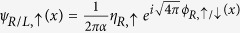, where *η*_*L*/*R*_ is the Klein factor to preserve the anticommutivity of the fermionic field which obeys the Clifford algebra^33^. Here we introduce the two bosonic fields, *θ*(*x*) and *ϕ*(*x*), which are dual to each other. These two fields are related with the following relations, *ϕ*(*x*) = *ϕ*_*R*_(*x*) + *ϕ*_*L*_(*x*) and *θ*(*x*) = *θ*_*R*_(*x*) + *θ*_*L*_(*x*). The analytical relation between the Klein factors have mentioned in ref. 33. The bosonized form for the Hamiltonian (*H*_01_) is,


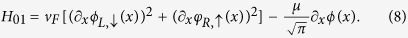


We use the relation, 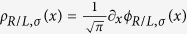, during the derivation of *H*_01_. Now we present the bosonized version of *H*_*f*_, *H*_*um*_, *H*_*B*_ and *H*_Δ_. We derive these analytical expressions by following the ref. 33, given by.


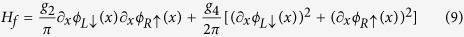



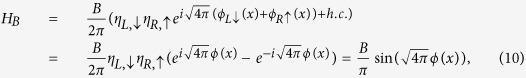



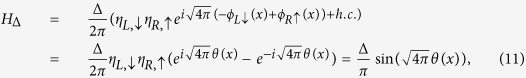



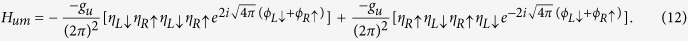


Finally from Eq. 12, we can write 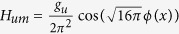, 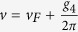, 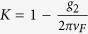. Explanation of sign mismatch with ref. 16 is the following: The author of ref. 16 have considered 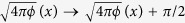, 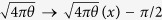.

### Renormalization Group Equations

Now we discuss the procedure for the derivation of RG equations, let us consider two operators, 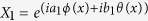 and 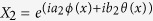. In the RG procedure, one can write these two field operators as a sum of fast and slow mode fields. In the fast field, the momentum range is Λ*e*^−*dl*^ < *K* < Λ and for the slow field *K* < Λ*e*^−*dl*^, where Λ is the momentum cut-off, *dl* is the change in the logarithmic scale. The next step is the integration of the fast field for the operators *X*_1_ and *X*_2_ and it yields a third operator at the same space time point, 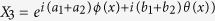. The prefactor of *X*_3_ can be found by the relation, 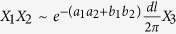. Our Hamiltonian will consist of two operators, if we consider *l*_1_ and *l*_2_ as the coefficients of the operators *X*_1_ and *X*_2_ respectively. Then the RG expressions for 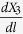 contain the term 
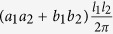
. This is the procedure to derive these RG equations of the present problem, which are the following:


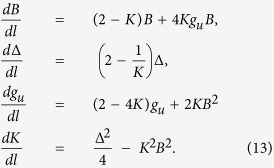


Where 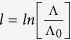, is the flow parameter and Λ_0_ is the initial value of the momentum cut-off parameter. In the RG process, one can write the RG equations themselves in a perturbative expansion in coupling constant (*g*(*l*)). They cease to be valid beyond a certain length scale, where *g*(*l*) ~ 1^34^. It is very clear from the above RG equations that in the absence of umklapp scattering, these equations reflect the duality in our helical liquid model system. The duality is the following: *ϕ* ↔ *θ*, *K* ↔ *K*^−1^ and Δ ↔ *B*. These RG equations have trivial (Δ^*^ = 0 = B^*^) fixed points for any arbitrary *K*. Apart from that, these RG equations have also two non-trivial fixed lines, Δ = *B* and Δ = −*B* for *K* = 1.

## Additional Information

**How to cite this article**: Sarkar, S. Physics of Majorana modes in interacting helical liquid. *Sci. Rep.* **6**, 30569; doi: 10.1038/srep30569 (2016).
